# P-1743. Plasma Metagenomic Next-generation Sequencing for Diagnosis of Invasive Fungal Infections in Children

**DOI:** 10.1093/ofid/ofaf695.1914

**Published:** 2026-01-11

**Authors:** Mario M Landa, Sonali Chaudhury, Jenna Rossoff, Ayelet Rosenthal, William J Muller

**Affiliations:** Ann & Robert H. Lurie Children's Hospital of Chicago, Chicago, IL; Lurie Childrens Hospital, Chicago, Illinois; Ann & Robert H. Lurie Children's Hospital of Chicago, Chicago, IL; Lurie Children's Hospital, Northwestern University, Chicago, Illinois; Ann & Robert H. Lurie Children's Hospital of Chicago, Chicago, IL

## Abstract

**Background:**

Invasive fungal infection (IFI) is challenging to diagnose, often involving invasive sampling. Plasma cell-free metagenomic next-generation sequencing (mNGS) has shown promise in diagnosing infections, but data are limited on specific clinical scenarios in which this test is most helpful. We investigated plasma mNGS testing for evaluation of children with high-risk immunocompromising conditions and clinical concern for IFI.

**Figure 1.**
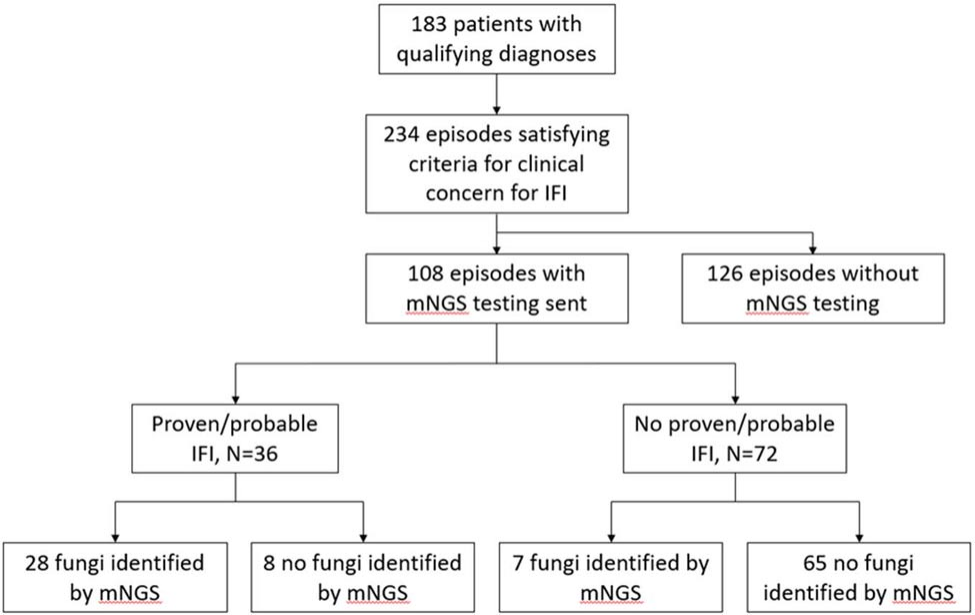
Study flowchart

**Table 1.**
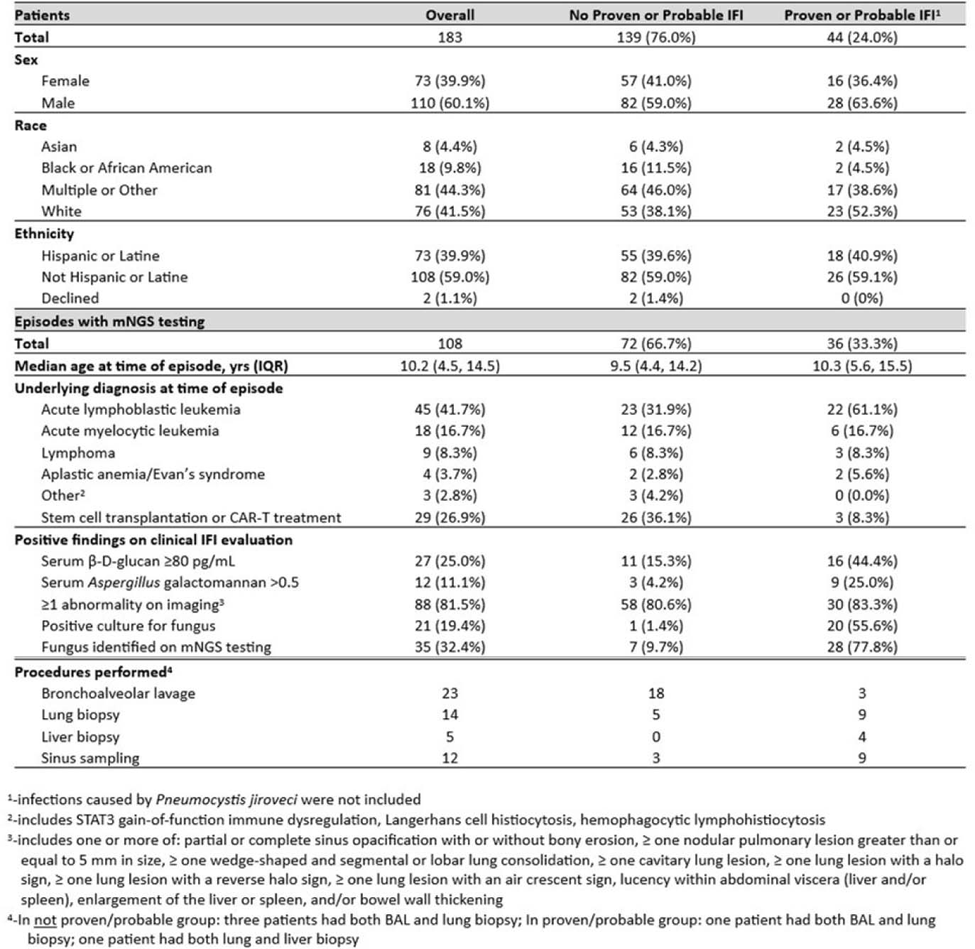
Characteristics of patients and episodes included in the study

**Methods:**

Retrospective single-center, case-control study of patients with high-risk conditions evaluated for IFI at Lurie Children’s between Dec 2016 and Sep 2024 (Table 1). Patients had a qualifying episode when criteria for clinical concern for IFI were met, including: (a) evaluation with both serum β-D-glucan and galactomannan testing, (b) either or both of CT scans of sinuses and chest, and (c) antifungal treatment was either started or broadened. Episodes in which mNGS testing was sent within 30 days of initiation or change in antifungal coverage were included. Test performance characteristics were determined for episodes of proven or probable IFI compared to those without proven or probable IFI.

**Table 2.**
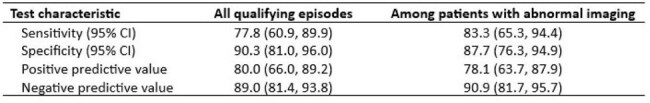
Performance characteristics of plasma mNGS testing for diagnosis of proven/probable IFI

**Table 3.**
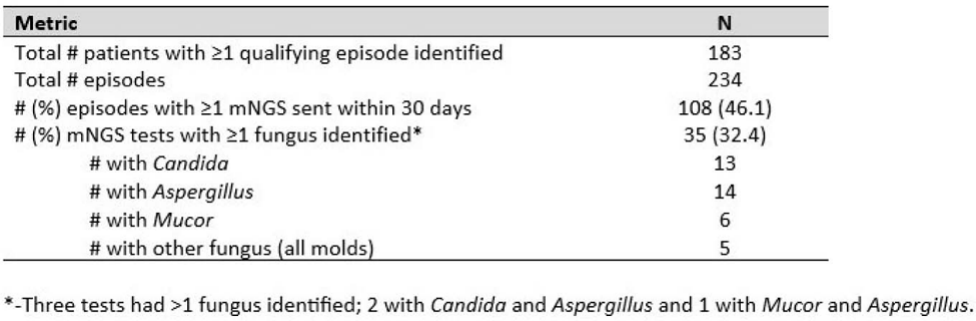
Characteristics of episodes with positive fungal identification on mNGS testing

**Results:**

A total of 183 patients experienced 234 episodes that met the criteria indicating clinical concern for IFI. Of 108 episodes in which plasma mNGS testing was sent, ≥1 fungus was identified in 35 (32.4%; Figure 1). Of 36 episodes which met EORTC-MSG criteria for proven/probable IFI in which plasma mNGS testing was sent, the test identified the causative organism in 28. Sensitivity and specificity for diagnosis of proven/probable IFI in this population was 77.8% and 90.3%, respectively, with positive and negative predictive value each above 80.0% (Table 2). Restricting the analysis to episodes with imaging abnormalities resulted in similar test performance characteristics. Among proven/probable cases with mNGS testing, *Candida* and *Aspergillus* were the most commonly identified fungi, identified in 13 and 14 episodes, respectively (Table 3).

**Conclusion:**

Plasma mNGS testing in pediatric patients at risk for IFI has favorable performance characteristics. Improving the identification of patients for whom testing is most sensitive and specific could lead to decreased need for invasive testing in some children.

**Disclosures:**

William J. Muller, MD, PhD, Ansun Biopharma: Grant/Research Support|Astellas Pharma: Advisor/Consultant|Astellas Pharma: Grant/Research Support|AstraZeneca: Advisor/Consultant|AstraZeneca: Grant/Research Support|Clarivate Analytics (US) LLC: Grant/Research Support|Eli Lilly and Company: Grant/Research Support|Enanta Pharmaceuticals: Advisor/Consultant|Enanta Pharmaceuticals: Grant/Research Support|F. Hoffmann-La Roche: Grant/Research Support|Finley Law Firm, P.C.: Advisor/Consultant|Gilead Sciences: Grant/Research Support|Melinta Therapeutics, Inc.: Grant/Research Support|Merck: Grant/Research Support|Moderna: Grant/Research Support|Nabriva Therapeutics, plc: Grant/Research Support|Paratek Pharmaceuticals, Inc.: Grant/Research Support|Pfizer: Grant/Research Support|Sanofi Pasteur LLC: Honoraria|Schueler, Dallavo, & Casieri: Advisor/Consultant|Tetraphase Pharmaceuticals, Inc.: Grant/Research Support|Vindico CME: Honoraria

